# Mediation by atherogenic index of plasma of the association between body roundness index and cognitive impairment in older Chinese people: an analysis using health check-up data

**DOI:** 10.3389/fpubh.2026.1851710

**Published:** 2026-06-19

**Authors:** Huaxing Wu, Binwei Wu, Juan Chen, Shangwei Liu, Minyong Ding, Manli Guan, Jintao Liu

**Affiliations:** 1 People’s Hospital of Jingning She Autonomous County, Lishui, China; 2 Chengzhao Township Health Center, Jingning She Autonomous County, Lishui, China; 3 Center for Disease Prevention and Control of Jingning She Autonomous County, Lishui, China

**Keywords:** atherogenic index of plasma, body roundness index, cognitive impairment, cross-sectional study, older adults

## Abstract

**Objective:**

The objective of this large sample cross-sectional study was to investigate the relationships between body roundness index (BRI), atherogenic index of plasma (AIP), and cognitive impairment in older Chinese people.

**Methods:**

This study included a population of 97,081 individuals who underwent health check-up at People’s Hospital of Jingning She Autonomous County from 2020 to 2024. Cognitive function was assessed using the Montreal Cognitive Assessment (MoCA). Data was collected through face-to-face interviews, physical examinations, and laboratory tests. BRI was calculated and grouped into quartiles. Univariate logistic regression models and multivariate logistic regression models were used for analysis. The restricted cubic spline (RCS) curve was used to investigate the dose–response relationship between BRI levels and cognitive impairment, and a mediation analysis was conducted to examine the role of AIP in mediating the effect of BRI on cognitive impairment.

**Results:**

The likelihood of cognitive impairment increased by 67.1% for each unit increase in AIP (OR = 1.671, 95%CI: 1.258–2.221, *p* < 0.001), and participants in the third quartile of BRI (Q3) had a 31.2% lower risk of cognitive impairment compared to those in the first quartile (Q1) (OR = 0.688, 95%CI: 0.528–0.897, *p* = 0.006). There was a nonlinear U-shaped relationship between BRI levels and cognitive impairment (P for nonlinear = 0.042), although the *p* value for overall was not statistically significant. Furthermore, AIP statistically accounted for the association between BRI and cognitive impairment (*p* < 0.001).

**Conclusion:**

Elevated AIP was independently associated with a higher risk of cognitive impairment in older Chinese adults, and statistically accounted for a substantial portion of the BRI–cognitive impairment association. The protective signal at intermediate BRI levels, without a linear trend, likely reflects a localized phenomenon rather than a global obesity paradox. Longitudinal studies are needed to confirm directionality.

## 1 Introduction

Cognitive impairment is a significant health concern among older adults, leading to a decline in life quality and even death (1, 2). Approximately 12 to 15% of older adults with mild cognitive impairment will develop irreversible dementia within 1 year (3). As the global aging population, the increase in cases of cognitive impairment has greatly increased the public and economic burden associated with healthy aging (4). According to epidemiological statistics, as of the end of 2020, there were over 40 million older adults aged 60 and above in China with mild cognitive impairment, and the number is increasing with the continuous growth of older adult population (5). Given these alarming trends, early identification and timely intervention are crucial for mitigating the risk and progression of cognitive impairment.

Lifestyle variables are often linked to cognitive impairment in older adults (6). A growing body of studies suggested that obesity was associated with an increased risk of cognitive impairment in older adults (7–9). Body Mass Index (BMI) was deemed as the major indicator to assessing obesity in these studies. Although BMI is a widely employed metric for body fat determined by height and weight, it lacks the ability to reflect body fat percentage and visceral adipose tissue (10). Visceral fat was considered more harmful than fat in other parts of the body, and even individuals with a normal BMI may have a significant accumulation of visceral fat (11). Moreover, the prognostic capability of BMI outside reference range was found to be hinged on anthropometric and clinical conditions (12). To solve these limitations, Thomas and his team introduced the body roundness index (BRI) through mathematical modeling to assess visceral fat levels (13). They developed elliptical models based on human body shape to calculate body roundness and used eccentricity to estimate visceral fat and total body fat percentages. Besides weight and height, BRI additionally considers waist circumference, enabling a more accurate evaluation of body fat and visceral fat distribution (14, 15). The previous study demonstrated BRI, in conjunction with age, gender, and hypertension, can serve as a useful predictor of cognitive impairment, particularly in younger populations (16). Moreover, a study based the data from the National Health and Nutrition Examination Survey (NHANES) collected from 2011 to 2014 found that elevated BRI was related to worse cognitive function in the older adult population (17).

It is worth noting that atherosclerosis and cognitive decline are age-related diseases, and they have similar risk factors (18). Numerous studies have found that lipid markers such as total cholesterol, triglycerides (TG), and high-density lipoprotein cholesterol (HDL-C) were associated with the risk of cognitive impairment (19–21). The atherogenic index of plasma (AIP) derived from the logarithmic ratio of TG to HDL-C has been recognized as an important marker of lipid metabolism (22). AIP was able to effectively, low-cost and quickly identify high-risk groups of cardiovascular disease events and predict the rapid progress of coronary atherosclerosis (23, 24). Zhou et al. (25) explored the relationship between AIP levels and cognitive impairment in 7,918 individuals aged 45 and older from the China Health and Retirement Longitudinal Study (CHARLS) and revealed that elevated AIP levels were linked to an increased risk of cognitive impairment in middle-aged and older adults, suggesting that managing dyslipidemia could help reduce this risk. In addition, recent evidence indicated that BRI and AIP were both independent risk factors for cardiovascular diseases (CVD), with AIP having a significant indirect association between BRI and CVD (26).

**Figure 1:**
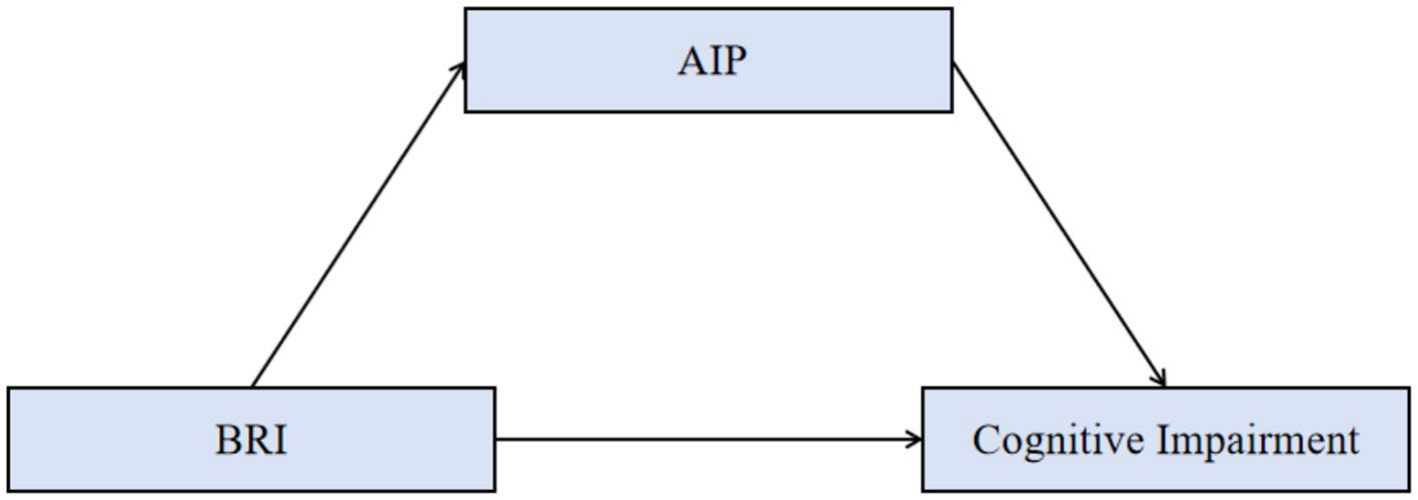
The hypothesis model of the relationships between BRI, AIP, and cognitive impairment.

Although BRI and AIP are independently associated with cognitive impairment, the potential mediating role of AIP between BRI and cognitive impairment in older adults has not been fully explored. In this context, this study utilized health check-up data to explore whether AIP mediated the association between BRI and cognitive impairment in older adults (Figure 1).

## 2 Materials and methods

### 2.1 Study population

**Figure 2:**
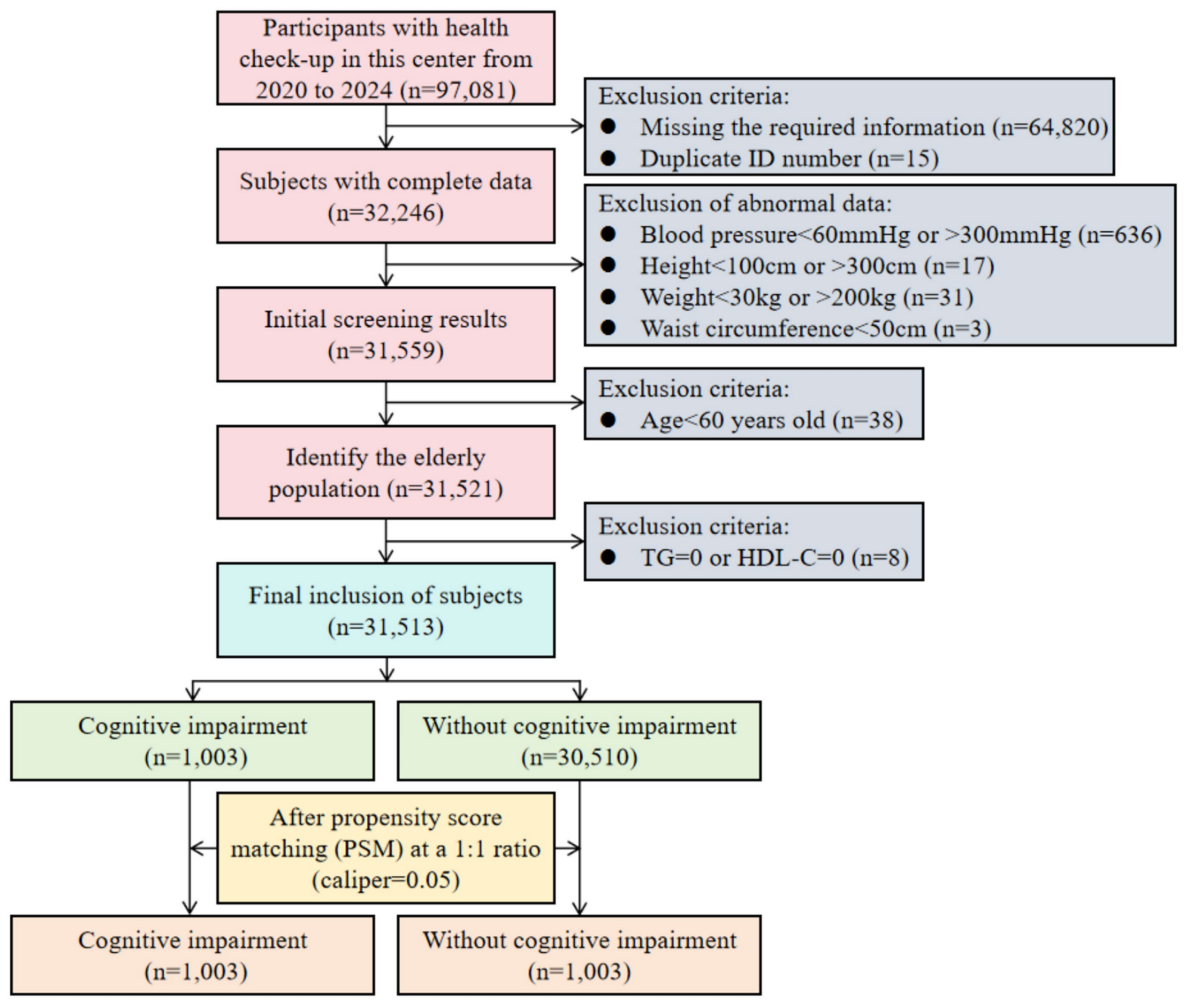
The flow chart of participants selection.

This study was a retrospective cross-sectional study that included a population of 97,081 individuals who underwent health check-up at People’s Hospital of Jingning She Autonomous County from 2020 to 2024. The flow chart of participants selection in this study was shown in Figure 2. A total of 31,513 subjects ultimately met the research criteria. All procedures performed in this study involving human participants were in accordance with the basic principles of the Declaration of Helsinki and had been approved by the Ethics Committee of People’s Hospital of Jingning She Autonomous County (approved number: 202508221658000320479). No informed consent was available due to the retrospective design.

### 2.2 Data collection

Age, gender (male/female), satisfaction with current life (fully satisfied/satisfied/basically satisfied/dissatisfied), self-care (totally/mild dependence on others/heavy dependence on others/cannot), exercise frequency (everyday/more than once a week/occasionally/never), dietary habits (meat-based diet/meat and vegetables/vegetarian diet), smoking (current smokers/past smokers/never), drinking alcohol (everyday/often/occasionally/never), and motor function (every action can be completed smoothly/unable to perform any of the actions) were collected by researchers through face-to-face interviews using questionnaire.

Besides, physical assessment was performed by physicians to obtain objective measurements, including systolic pressure, diastolic pressure, height, weight, and waist circumference. Height was recorded with the participant standing upright, and weight was measured with a standard scale. BMI was calculated as the weight in kilograms divided by the square of height in meters. We divided the performance of BMI using the World Health Organization reference standard into underweight: BMI < 18.5, normal: 18.5 ≤ BMI < 25, overweight: 25 ≤ BMI < 30, and obesity: BMI ≥ 30 (27). Waist circumference was measured in the standing position at the level of the umbilicus to the nearest 1 cm using a constant tension tape. For laboratory data, we obtained TG and HDL-C.

### 2.3 Cognitive impairment assessment

The Montreal Cognitive Assessment (MoCA) was used to assess the cognitive function, with scores ranging from 0 to 30. A total score of 25 or lower was indicative of the presence of cognitive impairment, and a normal range for cognition function was defined as a score of 26 to 30 (28).

### 2.4 Assessment of BRI

The formula for calculating BRI is as follows (13): BRI = 364.2–365.5 × √(1-[waist circumference in centimeters/2π]^2^/[0.5 × height in centimeters]^2^). The BRI was categorized into four levels in this study according to the 25th, 50th, and 75th quantiles. A higher BRI indicated a greater accumulation of visceral fat.

### 2.5 Assessment of AIP

The calculation of AIP was predicated upon the measurement on concentrations of TG and HDL-C in the blood, and the specific formula was presented as follows: AIP = log10[TG (mmol/L)/HDL-C (mmol/L)] (29).

### 2.6 Statistical analysis

The Kolmogorov–Smirnov test was used to determine the normality of variables distributions. Continuous variables were presented as mean ± SD or median (quartile distance), while categorical variables were presented as numbers (percentage). The t-test or Mann–Whitney *U* test was employed to analyze continuous variables, and categorical variables were compared using the chi-square test or Fisher exact test. Univariate logistic regression models and multivariate logistic regression models were performed to explore the relationships between demographic characteristics and cognitive impairment. Furthermore, propensity score matching (PSM) with a caliper value of 0.01 and a 1:1 ratio was utilized to minimize the influence of biases and confounding variables in this study. The restricted cubic spline (RCS) curve was also used to explore the dose–response relationship between BRI levels and cognitive impairment, with the median BRI value serving as the reference for comparison. Subsequently, the mediation analyses were conducted to ascertain the potential of the AIP to serve as a mediating factor between the BRI and cognitive impairment, and the confidence interval of the mediation effect was evaluated with the Bootstrap method to quantify the proportion of AIP in the mediation effect. To assess the robustness of the PSM main analysis against residual imbalance and to expand the set of adjusted covariates, we conducted an inverse probability of treatment weighting (IPTW) sensitivity analysis on the full eligible cohort. Covariate balance was assessed via standardized mean differences (SMDs), with absolute values below 0.1 considered acceptable. Moreover, stabilized average treatment effect (ATE) weights were derived. The statistical analysis was conducted in R software version 4.4.3, and *p* < 0.05 was regarded to indicate statistical significance.

## 3 Results

### 3.1 Characteristics of the study population

Thirty-one thousand five hundred thirteen participants were ultimately enrolled in this study, with the median age was 74 (71, 79) years, of which 52.10% were male, 4.20% more than female. The BMI of most participants was assessed as normal, accounting for 65.44%. There were 30,510 participants who possessed normal cognition, and 1,003 participants possessed cognitive impairment. According to BRI values, participants were stratified into four groups (Q1-Q4), and detection rates about cognitive impairment in each group of 3.30, 3.18, 2.74, and 3.52%, respectively. In addition, the median AIP values were −0.11 (−0.29, 0.09) in Q1, 0.02 (−0.18, 0.23) in Q2, 0.10 (−0.09, 0.32) in Q3, and 0.15 (−0.04, 0.36) in Q4. The baseline characteristics of participants across different groups were shown in Table 1. No statistically significant difference was recorded between four groups just in motor function (*p* > 0.05).

**Table 1:** Characteristic information of the participants.

Variables	All (*n* = 31,513)	BRI categories in IQR				*P*
		Q1 (*n* = 7,941)	Q2 (*n* = 7,842)	Q3 (*n* = 7,951)	Q4 (*n* = 7,779)	
Gender						<0.001
Male	16,417 (52.10)	5,753 (72.45)	4,743 (60.48)	3,826 (48.12)	2,095 (26.93)	
Female	15,096 (47.90)	2,188 (27.55)	3,099 (39.52)	4,125 (51.88)	5,684 (73.07)	
Age (years)	74 (71,79)	74 (71,79)	74 (70,79)	74 (70,79)	74 (71,80)	<0.001
Systolic pressure (mmHg)	136 (125,149)	132 (120,144)	136 (125,149)	137 (127,150)	140 (130,152)	<0.001
Diastolic pressure (mmHg)	80 (73,86)	78 (71,84)	79 (73,85)	80 (74,86)	81 (74,87)	<0.001
BMI						<0.001
Underweight	1,769 (5.61)	1,557 (19.61)	176 (2.24)	30 (0.38)	6 (0.08)	
Normal	20,622 (65.44)	6,327 (79.67)	7,118 (90.77)	5,321 (66.92)	1,856 (23.85)	
Overweight	8,227 (26.11)	56 (0.71)	548 (6.99)	2,588 (32.55)	5,035 (64.73)	
Obesity	895 (2.84)	1 (0.01)	0 (0.00)	12 (0.15)	882 (11.34)	
Waist circumference (cm)	82 (76,88)	72 (69,76)	80 (77,83)	85 (82,88)	92 (88,96)	<0.001
Satisfaction with current life						0.006
Fully satisfied	25,022 (79.40)	6,284 (79.13)	6,233 (79.48)	6,311 (79.37)	6,194 (79.62)	
Satisfied	6,297 (19.98)	1,625 (20.46)	1,568 (19.99)	1,586 (19.95)	1,518 (19.51)	
Basically satisfied	150 (0.48)	22 (0.28)	33 (0.42)	38 (0.48)	57 (0.73)	
Dissatisfied	44 (0.14)	10 (0.13)	8 (0.10)	16 (0.20)	10 (0.13)	
Self-care						<0.001
Totally	31,281 (99.26)	7,901 (99.50)	7,809 (99.58)	7,876 (99.06)	7,695 (98.92)	
Mild dependence on others	161 (0.51)	26 (0.33)	21 (0.27)	54 (0.68)	60 (0.77)	
Heavy dependence on others	54 (0.17)	9 (0.11)	9 (0.11)	17 (0.21)	19 (0.24)	
Cannot	17 (0.05)	5 (0.06)	3 (0.04)	4 (0.05)	5 (0.06)	
Exercise frequency						<0.001
Everyday	5,589 (17.74)	1,068 (13.44)	1,431 (18.25)	1,573 (19.78)	1,517 (19.50)	
More than once a week	1,228 (3.89)	249 (3.14)	242 (3.09)	353 (4.44)	384 (4.94)	
Occasionally	989 (3.14)	188 (2.37)	244 (3.11)	254 (3.19)	303 (3.89)	
Never	23,707 (75.23)	6,436 (81.05)	5,925 (75.55)	5,771 (72.58)	5,575 (71.67)	
Dietary habits						<0.001
Meat-based diet	961 (3.05)	239 (3.01)	217 (2.76)	254 (3.19)	251 (3.23)	
Meat and vegetables	22,179 (70.38)	5,420 (68.25)	5,551 (70.79)	5,619 (70.67)	5,589 (71.84)	
Vegetarian diet	8,373 (26.57)	2,282 (28.74)	2,074 (26.45)	2,078 (26.14)	1,939 (24.93)	
Smoking						<0.001
Current smokers	6,758 (21.44)	2,787 (35.10)	1,946 (24.82)	1,365 (17.17)	660 (8.48)	
Past smokers	1,584 (5.03)	540 (6.80)	447 (5.70)	393 (4.94)	204 (2.62)	
Never	23,171 (73.53)	4,614 (58.10)	5,449 (69.48)	6,193 (77.89)	6,915 (88.89)	
Drinking alcohol						<0.001
Everyday	4,891 (15.52)	1,622 (20.43)	1,428 (18.21)	1,145 (14.40)	696 (8.95)	
Often	1,575 (5.00)	415 (5.23)	455 (5.80)	396 (4.98)	309 (3.97)	
Occasionally	3,452 (10.95)	921 (11.60)	813 (10.37)	886 (11.14)	832 (10.70)	
Never	21,595 (68.53)	4,983 (62.74)	5,146 (65.62)	5,524 (69.48)	5,942 (76.38)	
Motor function						0.503
Every action can be completed smoothly	31,437 (99.76)	7,923 (99.77)	7,827 (99.81)	7,932 (99.76)	7,755 (99.69)	
Unable to perform any of the actions	76 (0.24)	18 (0.23)	15 (0.19)	19 (0.24)	24 (0.31)	
AIP	0.04 (−0.16,0.26)	−0.11 (−0.29,0.09)	0.02 (−0.18,0.23)	0.10 (−0.09,0.32)	0.15 (−0.04,0.36)	<0.001
Cognitive impairment						0.041
With	1,003 (3.18)	262 (3.30)	249 (3.18)	218 (2.74)	274 (3.52)	
Without	30,510 (96.82)	7,679 (96.70)	7,593 (96.82)	7,733 (97.26)	7,505 (96.48)	

IQR, interquartile range.

### 3.2 Comparison of characteristics between the normal cognition group and the cognitive impairment group before and after PSM

Before matching, the baseline characteristics of age (*p* < 0.001), systolic pressure (p < 0.001), satisfaction with current life (p < 0.001), self-care (p < 0.001), exercise frequency (p < 0.001), dietary habits (p < 0.001), AIP (*p* = 0.017), and BRI (*p* = 0.041) were significantly different between two groups. After PSM using scoring items, including gender, age, BMI, systolic pressure, and diastolic pressure as factors, a new dataset of 2,006 participants was generated with 1,003 participants in each group. Cognitive impairment was significantly different in satisfaction with current life (*p* < 0.001), dietary habits (*p* < 0.001), and smoking (*p* = 0.002) after matching (Table 2).

**Table 2:** Comparison of characteristics between the two groups before and after PSM.

Variables	Before PSM Cognitive impairment		*P*	After PSM Cognitive impairment		*P*
	With (*n* = 1,003)	Without (*n* = 30,510)		With (*n* = 1,003)	Without (*n* = 1,003)	
Gender			0.821			0.592
Male	519 (51.74)	15,898 (52.11)		519 (51.74)	506 (50.45)	
Female	484 (48.26)	14,612 (47.89)		484 (48.26)	497 (49.55)	
Age (years)	75 (71,81)	74 (70,79)	<0.001	75 (71,81)	75 (71,81)	0.521
Systolic pressure (mmHg)	139 (128,152)	137 (126,149)	<0.001	139 (128,152)	138 (127,150)	0.230
Diastolic pressure (mmHg)	80 (73,87)	80 (73,86)	0.631	80 (73,87)	79 (73,86)	0.131
BMI			0.059			0.918
Underweight	51 (5.08)	1,718 (5.63)		51 (5.08)	48 (4.79)	
Normal	644 (64.21)	19,978 (65.48)		644 (64.21)	659 (65.70)	
Overweight	266 (26.52)	7,961 (26.09)		266 (26.52)	255 (25.42)	
Obesity	42 (4.19)	853 (2.80)		42 (4.19)	41 (4.09)	
Satisfaction with current life			<0.001			<0.001
Fully satisfied	611 (60.92)	24,411 (80.01)		611 (60.92)	804 (80.16)	
Satisfied	386 (38.48)	5,911 (19.37)		386 (38.48)	189 (18.84)	
Basically satisfied	3 (0.30)	147 (0.48)		3 (0.30)	7 (0.70)	
Dissatisfied	3 (0.30)	41 (0.13)		3 (0.30)	3 (0.30)	
Self-care			<0.001			0.052
Totally	984 (98.11)	30,297 (99.30)		984 (98.11)	995 (99.20)	
Mild dependence on others	10 (0.99)	151 (0.49)		10 (0.99)	7 (0.70)	
Heavy dependence on others	7 (0.70)	47 (0.15)		7 (0.70)	1 (0.10)	
Cannot	2 (0.20)	15 (0.05)		2 (0.20)	0 (0.00)	
Exercise frequency			<0.001			0.099
Everyday	142 (14.16)	5,447 (17.85)		142 (14.16)	168 (16.75)	
More than once a week	18 (1.79)	1,210 (3.97)		18 (1.79)	30 (2.99)	
Occasionally	45 (4.49)	944 (3.09)		45 (4.49)	39 (3.89)	
Never	798 (79.56)	22,909 (75.09)		798 (79.56)	766 (76.37)	
Dietary habits			<0.001			<0.001
Meat-based diet	49 (4.89)	912 (2.99)		49 (4.89)	35 (3.49)	
Meat and vegetables	815 (81.26)	21,364 (70.02)		815 (81.26)	706 (70.39)	
Vegetarian diet	139 (13.85)	8,234 (26.99)		139 (13.85)	262 (26.12)	
Smoking			0.077			0.002
Current smokers	237 (23.63)	6,521 (21.37)		237 (23.63)	178 (17.75)	
Past smokers	59 (5.88)	1,525 (5.00)		59 (5.88)	49 (4.88)	
Never	707 (70.49)	22,464 (73.63)		707 (70.49)	776 (77.37)	
Drinking alcohol			0.190			0.715
Everyday	164 (16.35)	4,727 (15.49)		164 (16.35)	150 (14.96)	
Often	59 (2.88)	1,516 (4.97)		59 (2.88)	52 (5.18)	
Occasionally	122 (12.16)	3,330 (10.91)		122 (12.16)	123 (12.26)	
Never	658 (65.60)	20,937 (68.62)		658 (65.60)	678 (67.60)	
Motor function			0.095			0.723
Every action can be completed smoothly	998 (99.50)	30,439 (99.77)		998 (99.50)	1,000 (99.70)	
Unable to perform any of the actions	5 (0.50)	71 (0.23)		5 (0.50)	3 (0.30)	
AIP	0.05 (−0.13,0.27)	0.04 (−0.16,0.26)	0.017	0.05 (−0.13,0.27)	0.05 (−0.14,0.25)	0.111
BRI			0.041			0.089
Q1	262 (26.12)	7,679 (25.17)		262 (26.12)	235 (23.43)	
Q2	249 (24.83)	7,593 (24.88)		249 (24.83)	235 (23.43)	
Q3	218 (21.73)	7,733 (25.35)		218 (21.73)	265 (26.42)	
Q4	274 (27.32)	7,505 (24.60)		274 (27.32)	268 (26.72)	

### 3.3 Relationships between BRI, AIP and cognitive impairment

**Figure 3:**
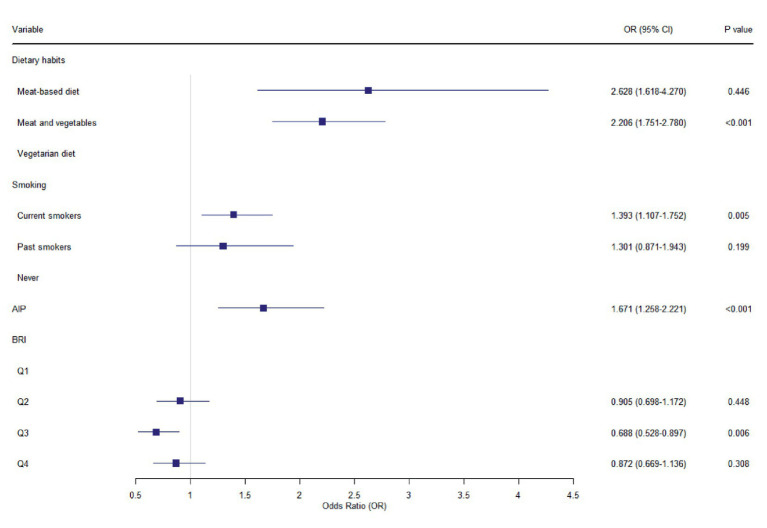
Relationships between BRI, AIP and cognitive impairment. 95%CI: 95% confidence interval.

**Figure 4:**
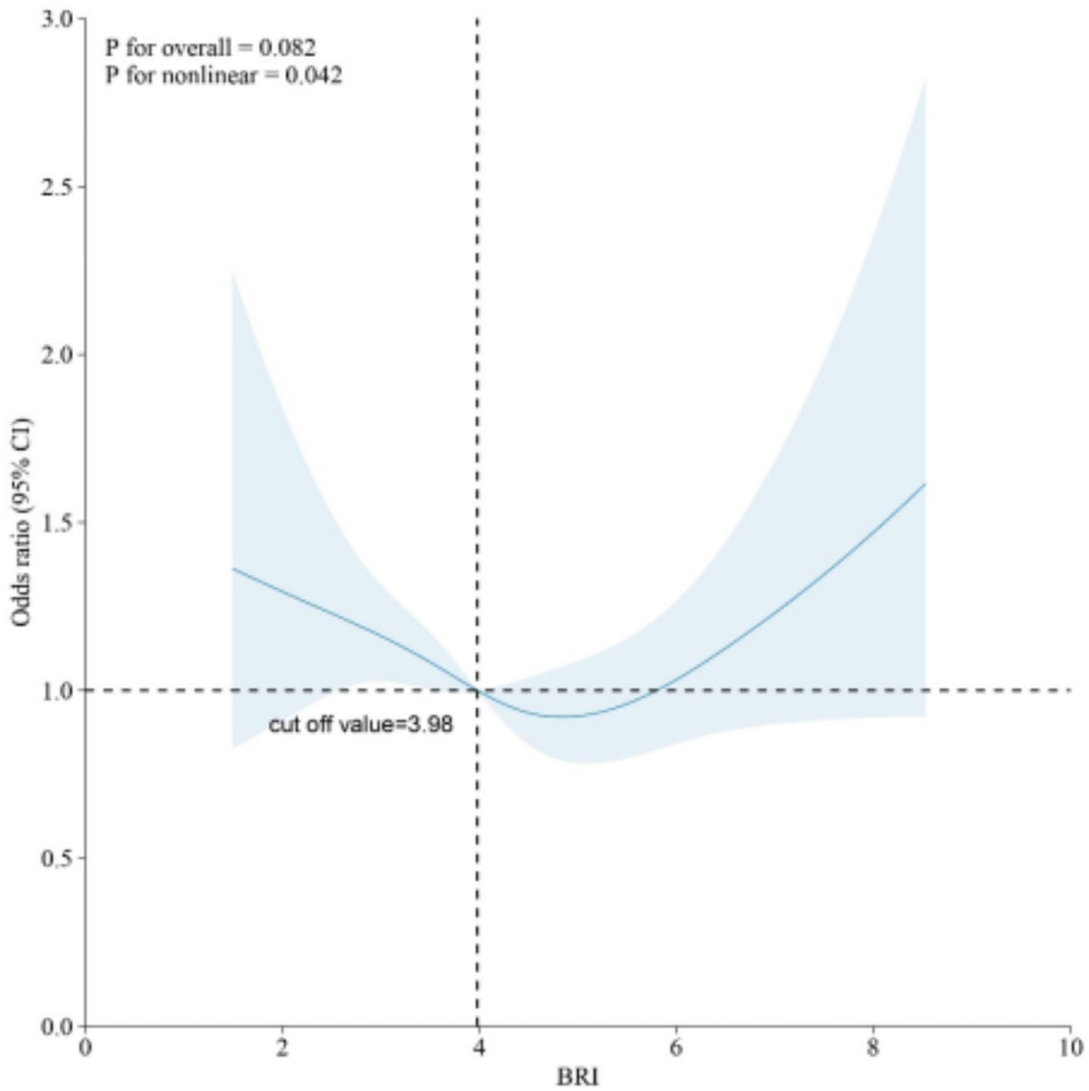
RCS analysis with multivariate-adjusted association between BRI and cognitive impairment.

Two thousand and six participants were included in the analysis of cognitive impairment risk. Univariable logistic analysis revealed that several factors, including dietary habits, smoking, AIP, and quartiles of BRI, were significantly associated with cognitive impairment (Table 3). In the multivariable logistic model, AIP and quartiles of BRI remained significantly associated with cognitive impairment after adjusting for lifestyle risk factors including dietary habits and smoking (Figure 3). In more detail, the results demonstrated that the likelihood of cognitive impairment increased by 67.1% for each unit increase in AIP (OR = 1.671, 95%CI: 1.258–2.221, *p* < 0.001). Additionally, this study found that participants in the third quartile of BRI (Q3) had a 31.2% lower risk of cognitive impairment compared to those in the first quartile (Q1) (OR = 0.688, 95%CI: 0.528–0.897, *p* = 0.006), and this association suggested that there may be a non-linear relationship between BRI and cognitive impairment. RCS analysis suggested a potential nonlinear pattern between BRI and cognitive impairment (P for nonlinear = 0.042), although the overall association did not reach statistical significance (*P* for overall = 0.082) (Figure 4). At representative BRI values (2, 3, 4, 5, 6, 7, 8) relative to the median, the odds ratios ranged from 0.92 to 1.47, with statistical significance observed only at BRI = 3 (OR = 1.162, 95%CI: 1.028–1.315) (Supplementary Table S1).

**Table 3:** The associated factors of cognitive impairment in the univariable logistic analysis.

Variables	Univariate logistic regression	
	OR (95%CI)	*P*
Gender		
Male	1.053 (0.884–1.255)	0.561
Female	Ref	
Age	0.997 (0.984–1.009)	0.618
Systolic pressure	1.003 (0.998–1.007)	0.269
Diastolic pressure	1.006 (0.997–1.016)	0.178
BMI		
Underweight	1.087 (0.722–1.636)	0.688
Normal	Ref	
Overweight	1.067 (0.871–1.308)	0.529
Obesity	1.048 (0.673–1.634)	0.835
Exercise frequency		
Everyday	0.811 (0.635–1.036)	0.094
More than once a week	0.576 (0.318–1.042)	0.068
Occasionally	1.108 (0.713–1.720)	0.649
Never	Ref	
Dietary habits		
Meat-based diet	2.639 (1.633–4.265)	<0.001
Meat and vegetables	2.176 (1.731–2.736)	<0.001
Vegetarian diet	Ref	
Smoking		
Current smokers	1.461 (1.173–1.820)	<0.001
Past smokers	1.322 (0.893–1.956)	0.164
Never	Ref	
Drinking alcohol		
Everyday	1.127 (0.881–1.441)	0.342
Often	1.169 (0.793–1.723)	0.430
Occasionally	1.022 (0.778–1.342)	0.875
Never	Ref	
AIP	1.490 (1.140–1.947)	0.004
BRI		
Q1	Ref	
Q2	0.950 (0.740–1.221)	0.691
Q3	0.738 (0.574–0.949)	0.018
Q4	0.917 (0.719–1.170)	0.486

95%CI, 95% confidence interval.

### 3.4 Mediation effect of AIP

In the mediation analysis, BRI was treated as the independent variable, cognitive impairment as the dependent variable, and AIP as the mediator. The results indicated that AIP statistically accounted for the association between BRI and cognitive impairment (*p* < 0.001), as detailed in Table 4.

**Table 4:** Mediation analysis of AIP in the association between BRI and cognitive impairment.

Type of effect	Estimate	95%CI Lower	95% CI Upper	*P*
Total effect	−0.004	−0.020	0.010	0.640
Average causal mediation effect	0.006	0.002	0.010	<0.001
Average direct effect	−0.010	−0.027	0.010	0.270
Prop mediated	−1.649	−9.233	8.061	0.640

95%CI, 95% confidence interval.

### 3.5 Sensitive analysis

To assess the stability of the observed associations against residual confounding, we conducted an IPTW sensitivity analysis. After weighting, all 31 covariate categories achieved adequate balance (all SMDs < 0.1; Supplementary Table S2 and Supplementary Figure S1), including the previously imbalanced life satisfaction (SMD reduced from 0.191 to 0.011), dietary habits (from 0.131 to 0.021), and systolic blood pressure (from 0.139 to 0.024). The IPTW-weighted models yielded effect estimates consistent with the PSM-based main analysis: AIP remained significantly associated with cognitive impairment (OR = 1.647, 95% CI: 1.317–2.060, *p* < 0.001), and participants in the third BRI quartile retained a lower risk relative to the first quartile (OR = 0.715, 95% CI: 0.569–0.900, *p* = 0.004). To further evaluate the BRI–cognitive impairment relationship, we tested BRI both as an ordinal trend variable across quartiles and as a continuous variable. Neither analysis reached statistical significance (P for trend = 0.135 in PSM, 0.122 in IPTW; continuous BRI in IPTW: OR = 0.971, 95%CI: 0.907–1.040, *p* = 0.406) (Supplementary Table S3).

## 4 Discussion

The objective of this large sample cross-sectional study was to investigate the relationships between BRI, AIP, and cognitive impairment in older Chinese people. In our study, the prevalence of cognitive impairment was 3.18%. The key findings revealed that dietary habits, smoking, AIP, and quartiles of BRI were significantly associated with cognitive impairment. After adjusting for health-related risk factors including dietary habits and smoking, our findings indicated that AIP was still significantly associated with a higher risk of cognitive impairment. Furthermore, individuals in the third BRI quartile demonstrated a lower risk of cognitive impairment than those in the first quartile. Importantly, the present study explored the potential mediating role of AIP between BRI and cognitive impairment in older Chinese people for the first time, and the mediation analysis revealed that AIP statistically accounted for the association between the BRI and cognitive impairment.

Previous studies have shown that lifestyle was closely related to cognitive impairment (30–32). In this study, compared to individuals with vegetarian diet, those with a habit consisting of meat and vegetables or meat-based diet had a higher risk of cognitive impairment. In mice, a fat-rich diet facilitated cognitive dysfunction. Wen et al. found by feeding 13-month-old C57BL/6 mice a normal or high-fat diet for 6 months that high-fat acid intake can hinder mitosis and upregulate Tau protein phosphorylation, including age-related synaptic dysfunction, leading to cognitive decline (33). Besides, epidemiological findings suggested that a high intake of saturated fats and cholesterol was associated with cognitive decline (34). However, variations in sample size and research methodologies across different regions can lead to inconsistent findings. A study based on the Chinese Longitudinal Healthy Longevity Survey indicated that compared with those who rarely/never consumed meat, participants consuming such product almost every day was 17% less likely to develop cognitive impairment (35). On the other hand, dietary diversity may offer a simple and straightforward method of identifying and screening individuals at high risk for cognitive impairment (36). Ding et al. (32) conducted the study among older adults residing in rural China showed that the healthy dietary pattern, which was based on the consumption of rice and flour, red meat, chicken, vegetables, seafood, and fruits, protecting against cognitive dysfunction. Furthermore, several studies also advocated for dietary diversity to alleviate cognitive decline and decrease the risk of cognitive impairment in older adults (37–39). At the same time, we found that smoking can negatively affect cognitive function. Numerous studies have shown a significant link between smoking and an increased risk of cognitive impairment, and these findings also supported our observation (40–42). Recently, research among 5,084 subjects aged 45 years and older similarly revealed that not smoking was a protective factor for cognitive function (43). Almeida et al. (44) found the reduced gray matter area in smokers may be a key area related to cognitive function, which was why smoking led to cognitive decline. China has the highest number of older adults with cognitive impairments in the world (45). Smoking is a modifiable risk factor, therefore community health service centers ought to adopt interventions for smokers to slow down cognitive impairment and reduce future social and economic burden.

There was still controversy over the relationship between measuring obesity-based BRI and cognitive impairment. A study conducted in a large Taiwanese cohort reported that increase in BRI connected with a decline in cognitive function (46). Conversely, Guo et al.’s (16) study indicated that obesity became a protective factor in certain health contexts. In response to this paradox, physiological explanations suggested that it was due to the survival advantage brought about by excessive fat storage (47). In this study, we found that participants in the third quartile of BRI had a lower risk of cognitive impairment compared to those in the first quartile, though this effect was not observed in the fourth quartile, in the absence of a significant linear trend or continuous-variable effect. It may reflect a threshold or localized phenomenon and requires replication in independent cohorts using longitudinal designs. Using the RCS model, we demonstrated a nonlinear U-shaped relationship between BRI levels and cognitive impairment, though the overall *p*-value was not statistically significant. This situation revealed an essential message that the relationship between BRI and cognitive impairment was complex, rather than simply the higher the worse or the lower the better. The rising and falling parts will offset each other to some extent, making the overall predictive ability of the variable appear less strong. On top of that, consistent with previous study (25), our findings demonstrated a significant positive correlation between elevated AIP levels and a higher risk of cognitive impairment. The underlying mechanisms linking AIP and cognitive impairment were complex and a widely accepted explanation was that elevated cholesterol levels may have a more direct effect on cognitive function (48). Notably, the current research indicated that AIP statistically accounted for the effects of BRI upon cognitive impairment. Given that both BRI and AIP are independent markers of cognitive impairment, it appears that AIP may serve as an intermediary, bridging the association between BRI and cognitive impairment.

Several limitations of the present study must be acknowledged. First, some confounding factors depend on self-reported data, such as satisfaction with current life, exercise frequency, and smoking. Although systematic errors were avoided as much as possible during the data collection process, there may remain information bias. Furthermore, important clinical variables and risk factors for cognitive impairment (e.g., drug use, medical treatment, education level, and health-related comorbidity factors) were not included in the analysis and therefore could not be adjusted for. The absence of these variables may have led to residual confounding. In particular, lower educational attainment is associated with both higher cardiometabolic risk and lower cognitive performance, which could partially explain the observed associations. Future studies incorporating these variables are warranted. Second, the participants in this study were all older adults aged 60 and above from People’s Hospital of Jingning She Autonomous County, and therefore further verification may be necessary to explore the applicability of these findings for other demographic groups. Third, the measurements of BRI and AIP were only in baseline, without considering the potential effects of changes in these indices over time on cognitive impairment. Fourth, the high exclusion rate raises concerned about selection bias, which could lead to underestimation of the true prevalence of cognitive impairment and potential attenuation of observed associations. Finally, cross-sectional design limited our ability to establish causal relationships between BRI, AIP, and cognitive impairment. The mediation results should be interpreted as exploratory and hypothesis-generating rather than as evidence of a causal pathway. Therefore, future studies should use longitudinal data or experimental designs to further validate these associations.

## 5 Conclusion

This large cross-sectional analysis provided evidence that elevated AIP is independently associated with a higher risk of cognitive impairment in older Chinese adults, and that AIP statistically accounts for a substantial portion of the BRI–cognitive impairment association. The observed protective signal at intermediate BRI levels, in the absence of a linear trend, is best interpreted as a localized phenomenon rather than a global obesity paradox. From a public health perspective, our findings suggested that monitoring plasma atherogenic burden may help identify older adults at higher cognitive risk, particularly within population-level health check-up programs. Longitudinal studies and mechanistic investigations are needed to confirm directionality and clinical relevance.

## Data Availability

The raw data supporting the conclusions of this article will be made available by the authors, without undue reservation.
