# Supplementary material for: Mediation by atherogenic index of plasma of the association between body roundness index and cognitive impairment in older Chinese people: an analysis using health check-up data
Source: Front Public Health. 2026 Jun 19;14:1851710. doi: 10.3389/fpubh.2026.1851710 (PMC13327974; doi:10.3389/fpubh.2026.1851710)
Supplement: Supplementary file 1 [file Table_1.DOCX]

**Supplementary Materials**

**Supplementary Table S1.** Adjusted odds ratios of cognitive impairment at representative body roundness index (BRI) values relative to the median BRI, derived from the restricted cubic spline model.

| **BRI** | **OR** | **95% CI** | **Statistical significance** |
| --- | --- | --- | --- |
| 2 | 1.293 | 0.910 – 1.837 | No |
| 3 | 1.162 | 1.028 – 1.315 | **Yes** |
| 4 | 0.997 | 0.993 – 1.001 | No |
| 5 | 0.922 | 0.783 – 1.086 | No |
| 6 | 1.030 | 0.840 – 1.264 | No |
| 7 | 1.228 | 0.899 – 1.678 | No |
| 8 | 1.467 | 0.917 – 2.345 | No |

Notes: Reference is the median BRI in the matched cohort. Odds ratios were estimated from a multivariable restricted cubic spline (RCS) logistic regression model with 4 knots, adjusted for dietary habits, smoking, drinking, and atherogenic index of plasma (AIP). P for overall = 0.082; P for nonlinear = 0.042. Only BRI = 3 reached statistical significance; the 95% confidence intervals at both ends crossed 1, indicating that the apparent U-shaped pattern is not robustly supported by point-wise inference.

**Supplementary Table S2.** Covariate balance before and after inverse probability of treatment weighting (IPTW).

| **Covariate** | **Type** | **SMD before IPTW** | **SMD after IPTW** |
| --- | --- | --- | --- |
| **Propensity score** | Distance | 0.5685 | **-0.0069** |
| Gender (male) | Binary | -0.0036 | 0.0070 |
| **Age** | Continuous | 0.2037 | **0.0621** |
| BMI category – underweight | Binary | -0.0055 | 0.0047 |
| BMI category – normal | Binary | -0.0127 | 0.0124 |
| BMI category – overweight | Binary | 0.0043 | -0.0171 |
| BMI category – obesity | Binary | 0.0139 | -0.0001 |
| **Systolic blood pressure** | Continuous | 0.1389 | **0.0244** |
| Diastolic blood pressure | Continuous | 0.0181 | 0.0062 |
| **Life satisfaction – fully satisfied** | Binary | -0.1909 | **0.0110** |
| **Life satisfaction – satisfied** | Binary | 0.1911 | **-0.0083** |
| Life satisfaction – basically satisfied | Binary | -0.0018 | -0.0029 |
| Life satisfaction – dissatisfied | Binary | 0.0016 | 0.0003 |
| **Dietary habits – vegetarian** | Binary | -0.1313 | **0.0206** |
| Dietary habits – meat-based | Binary | 0.0190 | -0.0007 |
| **Dietary habits – meat and vegetables** | Binary | 0.1123 | **-0.0199** |
| Smoking – never | Binary | -0.0314 | -0.0096 |
| Smoking – current | Binary | 0.0226 | 0.0065 |
| Smoking – past | Binary | 0.0088 | 0.0031 |
| Drinking – never | Binary | -0.0302 | 0.0068 |
| Drinking – everyday | Binary | 0.0086 | -0.0052 |
| Drinking – often | Binary | 0.0091 | -0.0004 |
| Drinking – occasionally | Binary | 0.0125 | -0.0012 |
| Exercise – everyday | Binary | -0.0370 | -0.0041 |
| Exercise – more than once a week | Binary | -0.0217 | 0.0045 |
| Exercise – occasionally | Binary | 0.0139 | 0.0024 |
| Exercise – never | Binary | 0.0447 | -0.0027 |
| Self-care – totally independent | Binary | -0.0120 | -0.0005 |
| Self-care – mild dependence | Binary | 0.0050 | 0.0007 |
| Self-care – heavy dependence | Binary | 0.0054 | -0.0000 |
| Self-care – cannot | Binary | 0.0015 | -0.0002 |

Notes: Standardized mean differences (SMDs) were computed by the cobalt package in R. All 31 covariate categories achieved SMDs below 0.1 after IPTW (balance threshold), indicating successful covariate balance. Stabilized average treatment effect (ATE) weights were applied, with the propensity score estimated using logistic regression on gender, age, BMI category, systolic and diastolic blood pressure, life satisfaction, dietary habits, smoking, drinking, exercise frequency, and self-care status.

**Supplementary Table S3.** Comparison of main effect estimates between the original propensity score matching (PSM) analysis and the inverse probability of treatment weighting (IPTW) sensitivity analysis.

| **Exposure** | **Analysis** | **OR (95% CI)** | **P value** |
| --- | --- | --- | --- |
| AIP (per unit increase) | PSM (main) | **1.671 (1.258 – 2.221)** | **<0.001** |
| AIP (per unit increase) | IPTW (sensitivity) | **1.647 (1.317 – 2.060)** | **<0.001** |
| BRI Q2 vs Q1 | PSM (main) | 0.905 (0.698 – 1.172) | 0.448 |
| BRI Q2 vs Q1 | IPTW (sensitivity) | 0.941 (0.758 – 1.168) | 0.583 |
| BRI Q3 vs Q1 | PSM (main) | **0.688 (0.528 – 0.897)** | **0.006** |
| BRI Q3 vs Q1 | IPTW (sensitivity) | **0.715 (0.569 – 0.900)** | **0.004** |
| BRI Q4 vs Q1 | PSM (main) | 0.872 (0.669 – 1.136) | 0.308 |
| BRI Q4 vs Q1 | IPTW (sensitivity) | 0.890 (0.707 – 1.121) | 0.322 |
| BRI (per unit increase, continuous) | PSM | 0.979 (0.911 – 1.052) | 0.570 |
| BRI (per unit increase, continuous) | IPTW | 0.971 (0.907 – 1.040) | 0.406 |
| BRI quartile (trend test) | PSM | 0.938 (0.862 – 1.020) | 0.135 |
| BRI quartile (trend test) | IPTW | 0.941 (0.871 – 1.016) | 0.122 |

Notes: All models adjusted for dietary habits and smoking. PSM estimates are from the main multivariable logistic regression model in the 1:1 propensity-matched cohort (n = 2,006). IPTW estimates are from weighted logistic regression with robust standard errors (survey package), applied to the full eligible sample (n = 31,513). The trend test and continuous BRI analyses confirm that the protective signal at BRI Q3 is non-monotonic and localized rather than reflective of a linear obesity paradox.

**Supplementary** **Figure S1.** Love plot showing covariate balance before (red circles) and after (blue triangles) inverse probability of treatment weighting (IPTW).


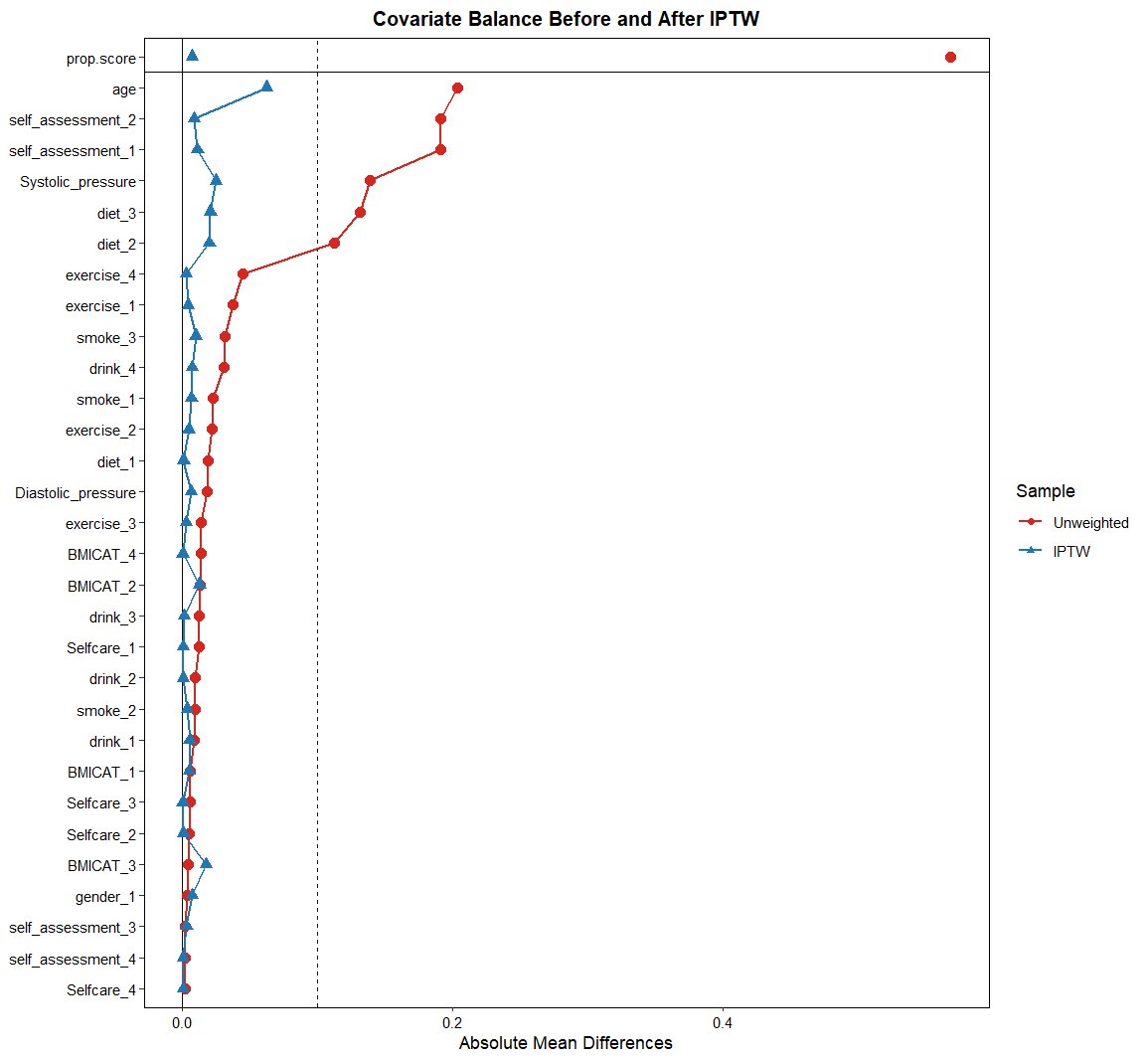


Notes: The x-axis shows absolute standardized mean differences (SMDs). The vertical dashed line indicates the conventional balance threshold of 0.1. Before IPTW (red), several covariates including the propensity score, age, life satisfaction, dietary habits, and systolic blood pressure exceeded the 0.1 threshold. After IPTW (blue), all covariates fell below 0.1, indicating adequate balance.
